# Serum Testosterone and Cortisol Concentrations After Single-Dose Administration of 100-Mg Transdermal Testosterone in Healthy Men

**DOI:** 10.3389/fphar.2019.01397

**Published:** 2019-11-21

**Authors:** Andrei A. Puiu, Sina Radke, Mikhail Votinov, Ute Habel, Beate Herpertz-Dahlmann, Bruce Turetsky, Kerstin Konrad

**Affiliations:** ^1^Child Neuropsychology Section, Department of Child and Adolescent Psychiatry, Psychosomatics and Psychotherapy, Faculty of Medicine, RWTH Aachen University, Aachen, Germany; ^2^Brain-Behavior Laboratory, Department of Psychiatry, Perelman School of Medicine, University of Pennsylvania, Philadelphia, PA, United States; ^3^Department of Psychiatry, Psychotherapy and Psychosomatics, Faculty of Medicine, RWTH Aachen University, Aachen, Germany; ^4^Institute of Neuroscience and Medicine: JARA-Institute Brain Structure Function Relationship (INM 10), Research Center Jülich, Jülich, Germany; ^5^Department of Child and Adolescent Psychiatry, Psychosomatics and Psychotherapy, Faculty of Medicine, RWTH Aachen University, Aachen, Germany; ^6^JARA-Brain Institute II Molecular Neuroscience and Neuroimaging, Research Centre Jülich, Jülich, Germany

**Keywords:** testosterone, transdermal, men, pharmacokinetics, cortisol, optimization

## Abstract

The growing interest in testosterone's effects on men's social behaviors, in particular aggressive, risk-taking, or status maintenance behaviors, is accompanied by a paucity of dose-dependent pharmacokinetic data. Examining the neurophysiological effects of transdermal testosterone typically includes a 4h delay before further brain-behavior measurements. Nevertheless, high heterogeneity regarding the timing of follow-up measurements and dosage remains. In a double-blind placebo-controlled design, we examined the short-term pharmacokinetic profile of 100-mg transdermal testosterone (Testotop^®^) to determine the optimal time for detecting testosterone-mediated effects. Across two studies, 35 healthy men received a single dose of testosterone and placebo in two separate sessions. In study one (n = 16), serum testosterone and cortisol were assessed serially every 30 min up to 2 h posttreatment. In study two (n = 19), we assessed serum testosterone and cortisol at baseline, 2 h, and 4.15 h (255 min) posttreatment. Relative to baseline and placebo, transdermal testosterone significantly increased total serum testosterone concentrations 90 min posttreatment, reaching maximum concentration between 2 h and 3 h posttreatment. Albeit elevated, serum testosterone levels gradually decreased between 2 h and 4 h following treatment. Transdermal testosterone did not suppress cortisol release. Instead, cortisol concentrations decreased according to cortisol's known circadian rhythm. Unlike previous findings showing significant testosterone concentration increases as soon as 60 min and as late as 3 h post 150-mg testosterone treatment, our 100-mg testosterone manipulation significantly increased testosterone concentrations 90 min following treatment. These pharmacokinetic data are important in facilitating the optimization of timing parameters for future testosterone challenge studies.

## Introduction

There is unprecedented interest in the role of sex-steroid testosterone (T) as a key modulator of men's social behaviors, in particular aggressive, risk-taking, or status maintenance behaviors. Due to its ability to quickly increase serum T levels above physiological ranges and its low potential for skin irritability, T gel preparations have become a popular agent not only in the treatment of hypogonadism but also in studies investigating the effects of T on human behavior. Transdermal T is absorbed into the systemic circulation bypassing the hepatic first-pass while mimicking the natural diurnal rhythm of testosterone release (Gooren and Bunck, 2003). Despite increasing interest in transdermal T due to its convenience and high tolerability, the paucity of pharmacokinetic data hinders the causal understanding of its neurophysiological effects.

A recent review on the pharmacokinetics of transdermal T preparations showed that different formulations restore T levels to pre-treatment concentrations of 14 ± 3.8 nmol/L as early as 24 h in men with normal physiological morning T levels (Arver et al., 2018). Plasma T concentrations return to pretreatment levels within the following 72 h. However, guidelines for defining atestosterone reference range are ill-defined. European consensus suggests that normal T levels range between 8 and 12 nmol/L (Wang et al., 2008). In the US, however, values below 7 nmol/L indicate hypogonadism, whereas values equal to or above 11.1 nmol/L are representative of normal physiological levels (Rosner et al., 2007). Moreover, these reference values vary considerably based on the assay kits used. For instance, across 25 laboratories, reference values for T assays ranged between 4.5 nmol/L and 14.6 nmol/L for the lower range and between 16.8 nmol/L and 40 nmol/L for the upper range (Lazarou et al., 2006). Nonetheless, commonly accepted values for the lower range hover around 8 nmol/L (± 1.6 nmol/L), whereas values of 29.5 ± 4.9 nmol/L indicate supra-physiological margins.

Approved dermal formulations include Testim^®^, Androgel^®^ (Testogel^®^), Tostrex^®^ (Fortesta^®^, Tostran^®^), Axiron^®^, Vogelxo^®^, Testavan^®^, and Testotop^®^. The bioavailability of testosterone from gel formulations varies between 10% and 15% (De Ronde, 2009), with median T_max_values reported between 18 h and 24 h (Marbury et al., 2003). In most hypogonadal men with morning T levels under 10.5 nmol/L, a single application of 50-mg or 100-mg Testim^®^ increased average serum T to a normal physiological range (Marbury et al., 2003; Steidle et al., 2003). Similarly, a 30-day regimen of daily 100-mg Testim^®^ administration increased baseline morning T concentrations from 8.7 nmol/L to 12.8 nmol/L (Dean et al., 2005) and improved men's sexual function and body composition up to 15 months following treatment. Reliable serum T increases in hypogonadal men are typically seen 3.5 h after transdermal administration (Marbury et al., 2003), with serum T levels experiencing up to a five-fold increases 24-h posttreatment followed by a plateau in the upper normal physiological range that gradually returns to pretreatment levels within the following 72 h (Wang et al., 2000).

Examining the effects of transdermal T typically includes a 4h delay before further assessments of brain-behavior effects. Nevertheless, high heterogeneity regarding both timing and dosage remains with some neurobehavioral studies implementing a 60-min time lag (100-mg Androgel^®^: Goetz et al., 2014), while others accounting for 120–180 min (150-mg Androgel^®^: Bird et al., 2016; 60-mg Tostrex^®^: Kopsida et al., 2016; Wu et al., 2018) or longer (50-mg Testim^®^: Panagiotidis et al., 2017; Wagels et al., 2017;Wagels et al., 2018; 50-mg Testogel^®^: Ortner et al., 2013).

A few studies to date have addressed the pharmacokinetics of transdermal T in healthy men. Relative to placebo, administering 50 mg of testosterone (Androgel^®^) to 12 healthy men increased serum T from a baseline average of 18.7 nmol/L to a peak average of 20.8 nmol/L 4h postadministration (Chik et al., 2006). Serum T concentrations gradually decreased within the following 2 h. Administering 100-mg topical Androgel^®^, however, to 25 men increased their total T and free T levels with 60% and 97%, respectively, 16h posttreatment (Zak et al., 2009). With 150-mg topical Androgel^®^ treatment, most studies reported significantly higher testosterone levels relative to baseline and placebo measurements 1h posttreatment; serum T levels remained elevated up to 2h posttreatment (Carré et al., 2015; Bird et al., 2016; Welling et al., 2016; Carré et al., 2017; Hansen et al., 2017). One study, however, showed that treating 10 men with a high dose of testosterone (150-mg Androgel^®^) increased serum T concentration to 39.7 nmol/L (relative to 18 nmol/L at baseline) 3 h following administration, which, albeit declining within 6h posttreatment (26.3 nmol/L), remained significantly above physiological values (Eisenegger et al., 2013). Transdermal T did not suppress cortisol, luteinizing hormone, or sex-hormone-binding globulin production. Unlike Chik et al. (2006) and Eisenegger and colleagues (2013)applied the treatment formulation on the left arm. Detailed treatment administration and rough treatment distribution information were unavailable. Previous findings showed that, when applied to different anatomical sites, the best absorption rates relative to skin thickness are obtained from the shoulders (suprascapular area) and thighs (Laurent et al., 2007; Guay et al., 2009).

A clean pharmacokinetic model of an acute single-dose 100-mg transdermal T preparation is currently missing. Therefore, we investigated whether the relationship between serum T concentrations following application of a commercially available transdermal T preparation (Testotop^®^) shows a similar linear relationship between dose and time as observed with smaller (50-mg Androgel^®^) and larger (150-mg Androgel^®^) dosages. Hence, in a fully randomized, double-blind, placebo-controlled study, we examined the short-term pharmacokinetic profile of the 100-mg Testotop^®^ preparation up to 2h posttreatment at 30-min intervals. In a second, identical study, we examined whether serum T concentrations stabilized between 2h and 4h posttreatment. The rationale for performing two separate studies was that we were interested in both short-term (first 2 h after administration) as well as in the longer-term (up to 4 h after treatment) time profile of transdermal testosterone as typical time delays observed in previous pharmacological challenge studies are heterogeneously chosen. However, considering bioethical regulations regarding biological samples (i.e., blood) collection for research purposes, serial blood sampling was limited to maximum five samples per subject.

## Methods

### Subjects and Study Design

Sixteen Caucasian men (M_age_= 22.4 ± 2.45 years old, two left-handed, BMI = 24.3 ± 3.38 kg/m^2^) participated in study one and nineteen men (all right-handed, M_age_= 23.8 ± 3.07 years old, BMI = 23.7 ± 2.33 kg/m^2^) took part in study two. All subjects were healthy and naïve to prior T administration upon participation in the fully-randomized within-subject experiments. Participants were recruited through media, on-campus advertisements, and an internal research database. We screened prospective participants for cardiovascular risk, preclinical signs of hypogonadism or hormonal dysfunctions, use of hormone or steroid supplements within one year before study participation, history of psychiatric disorders or neurological insult, irregular sleep patterns, use of recreational drugs within 6 months before study intake, habitual smoking, and drinking. Written informed consent was obtained on both study days and the study protocol was approved by the local ethical committee of the Medical Faculty of the RWTH University in accordance with the declaration of Helsinki. Participants received financial compensation upon completion of the second session.

### Testosterone Administration

Treatment administration was fully randomized according to a crossover, placebo-controlled, repeated-measures design. Participants received one 4-g tube containing 100-mg testosterone (Testotop^®^, Galenpharma GmbH, Wittland, Kiel, Germany) and another containing 100-mg placebo gel across two separate sessions. The testosterone sample contained additional ethanol (96%) as a testosterone solvent for aiding transdermal penetration, polyacrylate (carbomer 980) and propylene glycol for viscosity control, trometamol, disodium EDTA, and purified water. The placebo was produced to exactly match Testotop^®^'s inactive ingredients; the lack of testosterone was the only difference between the gels. Both treatments were odourless and colourless and were administered by a male investigator on the intact, dry skin of the upper part of the shoulders and the back of the neck in a roughly equal left-right distribution. Participants were instructed not to touch the area where the gel was administered and were allowed to dress again 30-min posttreatment. All participants responded to the transdermal T treatment. To account for belief effects, participants and the experimenter were asked to indicate which treatment they thought was administered at the end of each session.

### Procedure

Participants were tested on two occasions with an interval of at least 6 days between sessions. Both sessions were identical. Participants were asked to abstain from caffeine and alcohol 72 h, as well as from eating 2 h, prior treatment. All sessions were standardized and scheduled between 8.30 and 11.30 and between 8.30 and 13.30 for study one and for study two, respectively. Participants did not eat throughout the sessions. 10 min after arrival, baseline blood pressure and heart rate were measured. Approximately 5 ml of blood was collected at T_0_(before testosterone administration) to establish a baseline measurement of serum T and cortisol (C) concentration. For study one, we collected four serial blood samples at 30-min intervals up to 2-h posttreatment (T_1_=30_min_, T_2_= 60 min, T_3_=90_min_, T_4_= 120 min). In total, we collected five blood samples. For study two, we collected a total of three blood samples: one at baseline, one at 2h (T_4_), and another at 4.15-h (T_5_) posttreatment. We administered the treatments according to earlier protocols (Chik et al., 2006; Eisenegger et al., 2013) that demonstrated single-dose increases in plasma T levels 3h postadministration followed by a plateau at high levels four to 6h posttreatment.

### Hormone Profiles

Testosterone and cortisol serum concentrations were analyzed by electrochemiluminescence immunoassays (ECLIA, Roche^®^ Diagnostics GmbH). For testosterone, the inter-assay coefficient was 2.4% with a lower detection limit of 0.09 nmol/L. Respective inter-assay data for cortisol was 3.8% with a lower detection limit of 0.054 µg/dl. Intra-assay coefficients for testosterone and cortisol were below 3%. All analyses were conducted under strict internal and external quality control at the Clinical Chemistry, Haematology, Virology, and Microbiology Laboratory Diagnostic Centre (LDZ) of the RWTH Aachen University Hospital.

### Statistical Analyses

To assess the pharmacokinetic mapping of the testosterone preparation, serum T and C data from all subjects entered a repeated-measures ANOVA (RMANOVA) with treatment and time as within-subject factors. The alpha criterion level of statistical significance for all analyses was set at = .001 a. If significant, we report the eta squared () to illustrate the effects' magnitude. Small, medium, and large effects are reflected in values of equal to .10, .25, and .4, which correspond to values of *η²*of .0099, .0588, and .1379, respectively (Cohen, 1973; Richardson, 2011). In cases of significant main effects, we ran *post hoc t-*tests corrected for multiple comparisons. We additionally computed the percent change value relative to baseline for serum T and C concentrations at each time point. Analyses were carried out using JASP (Version 0.9).

## Results

### Testosterone Blood Serum Concentrations

#### Study 1 (n = 16)

**Figure 1:**
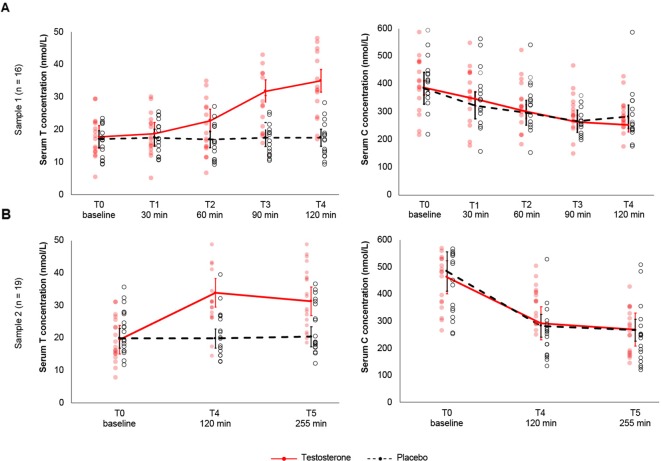
**(A)**. Study 1. Sex hormone testosterone and cortisol concentrations following transdermal T and placebo across 5 time-points and 16 subjects. Application of 100-mg transdermal T increased total serum T levels significantly relative to placebo and pretreatment as early as 90-min posttreatment. Serum T increased gradually to a maximum observed concentration 2h posttreatment. Testosterone levels in the placebo group (dashed black line) remained constant throughout the timely progression of the experiment. Serum C concentrations decreased with time independent of transdermal T, according to cortisol's independent circadian rhythm. Cortisol levels did not differ significantly between transdermal T and placebo treatment. **(B)**. Study 2. Sex hormone testosterone and cortisol concentrations following transdermal T and placebo across 3 time-points and 19 subjects. Serum T concentrations were significantly elevated 2h posttreatment. Although significantly higher relative to baseline and placebo treatment, 255-min posttreatment, serum T levels gradually decreased compared to 2h posttreatment. Serum C levels decreased independent of transdermal T. Whiskers indicate 99% confidence intervals. Red-filled and black dots represent the observed scores for the T and placebo treatments, respectively.

The RMANOVA revealed significant effects of treatment (*F*_1,15_= 28.5, *p*= .00008, *η²*= .166) and time (*F*_1.5,60_= 37.6, *p*< .00001, *η²*= .133), as well as a significant treatment-by-time interaction effect (*F*_1.6,25.3_= 36.8 *p* < .00001, *η²*= .124), indicating that T levels change differentially across time with transdermal T relative to placebo treatment (see **Figure 1A**). Subsequent treatment contrasts revealed a significant 7.92 nmol/L increase following transdermal T compared to placebo, *t*(15) = 5.33, *p* *_Bonferroni_* *= .*00008, Cohen's *d*= 1.33 (M_T_= 25.21 ± 9.17 nmol/L, M_PLC_= 17.29 ± 5.2 nmol/L). Serum T concentrations ranged between 17.7 nmol/L and 35 nmol/L following transdermal T. The maximum concentration was observed 2 h after transdermal T, and levels at this time point were significantly higher than those obtained 2 h after transdermal placebo, *t*(34.5) = 9.37, *p* *_Bonferroni_*< .00001. Baseline T levels between the two sessions did not differ significantly, *t*(15) = .65, (*p > .001)*. Relative to baseline, serum T concentrations increased 5.5% after 30 min, 28.3% after 60min, 79.1% after 90 min, and 97.2% after 120 min following treatment.

Serum T concentration did not differ significantly between transdermal T and placebo at baseline, 30-min, and 60-min posttreatment. Relative to placebo, serum T levels increased significantly 90-min and 120-min posttreatment [M_90min_= 14.7 nmol/L, *t*(15) = 5.7, *p*= .00004, *Cohen's d*= 1.44; M_120min_= 17.2 nmol/L, *t*(15) = 7.7, *p*< .00001, *Cohen's d*= 1.94] (**Figure 1A**). Compared to baseline concentrations (M_baseline_= 17.7 ± 6.7 nmol/L), transdermal T did not significantly increase serum T levels 30- or 60-min posttreatment M_30min_= 18.7 ± 6.9 nmol/L; M_60min_= 22.7 ± 8.7 nmol/L)^1^. Following transdermal T, testosterone levels increased between 30- and 90-min [M_30-90_= 13.1 nmol/L, *t*(15) = 5.9, *p*= .00003, *Cohen’s d* = 1.47], between 30- and 120-min [M_30-120_= 16.2 nmol/L, *t*(15) = 8.2, *p* < .00001, *Cohen’s d* = 2.05], between 60- and 90-min [M_60-90_= 9.01 nmol/L, *t* (15) = 7.9, *p* < .00001, *Cohen’s* *d* = 1.97], and between 60- and 120-min posttreatment [M_60-90_= 12.2 nmol/L, *t*(15) = 10, *p*< .00001, *Cohen's d*= 2.50]. Serum T concentration did not differ significantly between 90- and 120-min posttreatment (M_90-120_= 3.29 nmol/L, *p*> .05).

#### Study 2 (n = 19)

The RMANOVA revealed significant effects of treatment (*F* *_1,18_*= 28.8, *p*= .00004, *η²*= .171) and time (*F* *_2,36_*= 21.9, *p*< .00001, *η²*= .102), as well as a significant treatment-by-time interaction (*F* *_2,36_*= 25.1, *p*< .00001, *η²* = .098). Relative to baseline concentrations, serum T increased 73.5% after 2 h and 60.5% after 4.15 h following transdermal T treatment. Compared to the results above, transdermal T increased serum T levels 8.19 nmol/L relative to placebo, *t*(18) = 5.37, p*_Bonferroni_*= .00004, Cohen's*d* = 1.23 (M_T_= 28.26 ± 9.12 nmol/L, M_PLC_= 20.07 ± 6.4 nmol/L) (**Figure 1B**). Baseline T concentrations did not differ between transdermal T and placebo,*t*(18) = -.48, (*p*> .05). Relative to baseline (M = 19.5 nmol/L), serum T concentrations were significantly higher 120-min and 255-min posttreatment [M_120min_= 33.9 nmol/L, *t*(71.4) = 9.05, *p*< .00001, Cohen's *d* = 1.32; M_255min_= 31.4, *t*(71.4) = 7.45, *p* < .00001, Cohen's *d* = 1.57). Relative to placebo, transdermal T increased serum T concentrations 13.9 nmol/L between baseline and 2-h posttreatment, *t*(46.5) = 6.92, *p* = .00001, 14.01 nmol/L 2-h posttreatment, *t*(40.6) = 7.14, *p* = .00001, and 13.4 nmol/L 255-min posttreatment, *t*(46.5) = 6.67, *p*= .00003.

### Cortisol Blood Serum Concentrations

#### Study 1 (n = 16)

We found a significant main effect of time on serum C concentrations (*F*_4,60_= 21.2, *p* < .00001, *η²* = .198), reflecting the known circadian fluctuation in cortisol secretion (**Figure 1B**). Neither the main effect of T treatment (*F*_1,15_= 0.000217, *p* = .98) nor the interaction between treatment and time (*F*_4,60_= 0.085, *p* = .*49*) reached significance. Posthoc tests showed that, relative to baseline, serum C decreased 86.9 nmol/L after 60 min [*t*(60) = 5.58, *p* *_Bonferroni_*< .00001, Cohen's d = 1.56], 120.6 nmol/L after 90 min [*t*(60) = 7.7, *p* *_Bonferroni_*< .00001, Cohen's d = 1.61], and 117.9 nmol/L after 120 min following transdermal T [*t*(60) = 7.5, *p* *_Bonferroni_*< .00001, Cohen's d = 1.24]. Moreover, serum C concentrations decreased 69.6 nmol/L between 30 and 90 min [*t*(60) = 4.4, *p* *_Bonferroni_* *= .*00034; Cohen's d = 1.32] and 67 nmol/L between 30 and 120 min following transdermal T [*t*(60) = 4.3, *p* *_Bonferroni_* *= .*00062, Cohen's d = 0.89] (**Table 1**). Relative to baseline, serum C concentrations decreased 10.3% after 30 min, 22% after 60 min, 31.8% after 90 min, and 34.7% after 120 min following transdermal T. Similarly, compared to baseline, transdermal placebo decreased serum C concentrations 16.2% after 30 min, 23% after 60 min, 30.8% after 90 min, and 26.5% after 120 min following treatment.

**Table 1:** Treatment by time serum T and C average concentrations.

Study	Time (min)	Treatment	Serum T avg.					Serum C avg.				
			nmol/L	SE	SD	99% CIs		nmol/L	SE	SD	99% CIs	
						Lower	Upper				Lower	Upper
Study 1 - n = 16	0	Testosterone	17.8	1.7	6.8	12.5	23.0	386.7	24.7	98.7	286.3	487.2
		Placebo	17.1	1.2	4.9	11.9	22.3	384.9	28.0	112.1	270.7	499.0
	30	Testosterone	18.7	1.7	6.9	13.5	24.0	347.1	22.5	90.0	255.4	438.7
		Placebo	17.5	1.3	5.2	12.3	22.7	322.6	27.0	107.9	212.8	432.4
	60	Testosterone	22.8	2.2	8.8	17.6	28.0	301.5	21.5	86.0	213.9	389.1
		Placebo	17.0	1.4	5.5	11.7	22.2	296.2	22.5	89.9	204.7	387.7
	90	Testosterone	31.8	3.0	12.2	26.6	37.0	263.8	18.5	74.1	188.3	339.2
		Placebo	17.4	1.2	4.8	12.2	22.6	266.5	27.6	110.3	154.2	378.8
	120	Testosterone	35.0	2.8	11.2	29.8	40.2	252.7	15.3	61.2	190.4	315.0
Study 2 - n = 19		Placebo	17.5	1.4	5.5	12.3	22.7	282.9	24.0	96.0	185.2	380.6
	0	Testosterone	19.5	1.3	5.8	14.6	24.5	462.2	20.9	93.7	405.6	518.9
		Placebo	19.9	1.3	5.9	14.9	24.9	483.6	25.9	116.1	427.0	540.2
	120	Testosterone	33.9	2.6	11.6	28.9	38.9	292.5	20.3	90.7	235.9	349.2
		Placebo	19.9	1.5	6.7	14.9	24.8	282.7	18.8	84.0	226.0	339.3
	255	Testosterone	31.4	2.2	10.0	26.4	36.3	267.9	15.2	68.0	211.3	324.6
		Placebo	20.4	1.5	6.7	15.4	25.4	266.3	22.0	98.5	209.6	322.9
SE, standard error; SD, standard deviation; CIs, confidence intervals.												

#### Study 2 (n = 19)

Similarly, the RMANOVA results yielded a significant main effect of time, *F*_2,36_= 62.7, *p*< .00001, *η²*= .51. Neither the main effect of treatment (*F*_1,18_= 0.065, *p = .*80) nor the treatment-by-time interaction (*F*_2,36_= 0.67,*p = .51*) reached significance. *Post hoc*tests showed that serum C levels decreased 185.3 nmol/L between baseline and 2h posttreatment, *t*(36) = 9.49, *p < .*00001, Cohen's*d*= 2.17, and 205.8 nmol/L between baseline and 255-min posttreatment, *t*(36) = 9.32, *p < .*00001, Cohen's *d*= 2.13. Serum C concentrations did not differ significantly between 2h and 255-min posttreatment, *t*(36) = 1.01 (*p*= .57). Relative to baseline, total serum C concentrations decreased 58% 2h following transdermal T and 71.1% following transdermal placebo. 4.15h posttreatment, serum C levels decreased 72.5% following transdermal T relative to 81.6% following transdermal placebo.

## Discussion

Here, we show the short-term pharmacokinetic profile of single-dose 100-mg transdermal T in healthy young men. Compared to baseline concentration and placebo application, serum T concentrations were significantly elevated 1.5 h and 2 h following transdermal T. Testosterone concentrations, however, did not differ significantly between 90- and 120-min following T treatment. Furthermore, in a second study, we observed the same timely progression of serum T increase following transdermal T at the 2h assessment. Serum T concentration, although gradually declining, remained significantly elevated relative to baseline concentration and placebo application at 255-min posttransdermal T. Moreover, testosterone administration did not suppress cortisol release. Serum C concentrations decreased gradually across the 2 h, reflecting cortisol’s normal circadian rhythm.

Testosterone and its metabolites alter cellular excitation through a complex genomic cascade of events. Briefly, testosterone binds to androgen receptors and steroid-binding molecules. The steroid-receptor complex then translocates to the nucleus, where steroids interact with hormone-responsive nucleotide sequences to silence or activate protein synthesis and gene expression (Tsai and O'Malley, 1994; Bailey and Silver, 2014). Activation of this genomic pathway, however, is lengthy. For aldosterone, for instance, early genes become differentially expressed 1 h following steroid manipulation (Verrey, 1998). Clinically, at the level of the entire organism, genomic effects have an onset latency of 2 to 12 h (Maggi et al., 2004). In contrast to the genomic pathway, recent evidence shows that nongenomic mediated phenomena often occur with a very short delay (Bennett et al., 2010; Shihan et al., 2014). For instance, intrahippocampal injection of testosterone coupled with a protein synthesis inhibitor that prevents genomic effects improved spatial memory in adult male rats (Naghdi et al., 2005; but see Kelly et al., 2014). This highlights the possibility that nongenomic effects may be antagonistic to genomic mechanisms. However, the former may parallel gene expression while preceding genomic actions by up to 4 h (Michels and Hoppe, 2008). Similarly, it has been proposed that within half an hour following androgen manipulations, effects are likely attributable to fast non-genomic mechanisms, whereas later genomic effects are the major molecular and physiological mediator (Celec et al., 2015). While the physiological relevance of genomic and non-genomic androgen actions is still elusive, evidence suggests that the delayed social and neurophysiological effects observed in steroid challenge experiments may be due to their genomic and non-genomic effects (Filova et al., 2015).

Growing evidence indicates that testosterone has a robust influence on socio-emotional processing (for a review, see Bos et al., 2012). Some studies found positive effects of T on spatial and working memory abilities (Janowsky et al., 2000; Cherrier et al., 2001; Hamson et al., 2016), while others showed detrimental (Maki et al., 2007) or no T effects whatsoever. There is consensus though that T has anxiolytic and antidepressant effects (Celec et al., 2015). However, the paucity of data on the kinetics of T *in vivo*co mplicates the interpretation of the findings above as parameters such as subjects' age, sex, or current endocrine status add to the complex interactions between T and the brain. Our data on the dose and timing of T administration may facilitate the optimization of timing parameters for future T challenge studies.

Here, we found maximum T concentrations 2h posttreatment, which coincide with previous pharmacokinetic data. However, we observed a sharp significant increase in serum T (relative to baseline and transdermal placebo) as early as 1.5h post transdermal T. Although serum T levels increased further after 120-min posttransdermal treatment, the difference between 90 and 120 min in serum T concentrations did not differ significantly. This indicates that, with 100-mg Testotop^®^, serum T levels have a sharp increase 1.5 h after transdermal T, followed by a plateau 30 min later. This finding is supported in a second study (n = 19) where serum T levels increased up to 2-h posttreatment followed by a steady decrease at the 4.15h measurement. Corroborating previous evidence (Chik et al., 2006; Eisenegger et al., 2013; Welling et al., 2016; Carré et al., 2017; Hansen et al., 2017), we tentatively suggest that the optimal behavioral testing window for examining causal 100-mg transdermal T neurophysiological effects lies between 1.5h and 4.5h posttreatment. Although serum T levels gradually declined 4 h following transdermal treatment, we expected them to remain significantly elevated relative to placebo and baseline for an additional 2 to 3 h (Eisenegger et al., 2013).

Transdermal T did not suppress serum C levels. Instead, serum C concentrations followed cortisol's known independent circadian rhythm. Although both steroids have a rhythmic circhoral pulse (secreted serially approximately every 90 min), the morning levels of cortisol, despite its individually variable rhythm, are four-/five-fold higher than those in the evening (Bailey and Silver, 2014). Secreted by the hypothalamic-pituitary-adrenal (HPA) axis in response to stress, cortisol inhibits the activity of the hypothalamic–pituitary–gonadal axis (HPG axis) and is antagonistic to testosterone, blocking androgen receptors (Viau, 2002; Liening and Josephs, 2010). Although the causal effect of T on stress reactivity warrants further research, rat and human stress models link T to reduced cortisol reactivity to stress (Viau and Meaney, 2004) and suppressed cortisol responses to exogenous stimulation of the HPA axis in men (Rubinow et al., 2005). Our findings do not support testosterone's inhibitory effects on cortisol release. Serum C concentrations decreased throughout the sessions independent of transdermal T administration. This might be because testosterone's inhibitory effects are likely modulated by psychosocial variables such as negative effect, threats to social status, or social ostracism (Josephs et al., 2006; Mehta et al., 2008; Zilioli and Watson, 2013). Alternatively, from a physiological perspective, it may be that a single transdermal T dose is insufficient in both magnitude and extent to elicit the high systemic androgen concentrations necessary to suppress the HPA axis (Eisenegger et al., 2013). Carefully designed pharmacological interventions are needed to parcel out the mixed evidence (Stephens et al., 2016) linking testosterone's effects to cortisol release.

While these results match previous findings, they are subject to some considerations. First, due to ethical limitations, only five serial blood samples could be drawn from each participant. To circumvent this, we ran the same protocol across two independent samples/studies. Additionally, although we lacked the 3h measurement as per Eisenegger and colleagues (2013), our findings are congruent with previous pharmacokinetic data (Welling et al., 2016; Carré et al., 2017; Hansen et al., 2017) insofar as we show a similar trend in total T and C concentrations. Second, as we did not measure sex-hormone binding globulin (SHBG) or albumin, we did not compute the free testosterone (FT) fraction. FT reflects the level and activity of bioactive testosterone activating the androgen receptors (Hammond et al., 2012). However, calculated FT is more informative for clinical practice where total testosterone values are significantly below the physiological range. The absence of FT from our design does not hinder the understanding of our proposed transdermal T model. Third, we used immunoassays to determine serum parameters. While immunoassaying might lack reliability and specificity compared to liquid or gas chromatography with mass spectrometry (Rautenberg and Lentjes, 2007) when determining a comprehensive andrological status (i.e., including oestrogen or dihydrotestosterone) in clinical populations (Hsing et al., 2007), immunoassays are fast and economical, and thus appropriate when assessing simple physiological parameters (i.e., total serum T, C) within an expected physiological range. Future pharmacokinetic studies should investigate drug volume of distribution (i.e., apparent volume in which a formulation is distributed), elimination half-life, or elimination rates to provide a comprehensive profile of the 100-mg Testotop^®^ formulation.

In conclusion, we examined the pharmacokinetic profile of a 100-mg transdermal T formulation and found that it quickly and significantly elevated total serum T levels 90-min posttreatment. Moreover, we found no inhibitory effects of transdermal T on serum C concentrations, the latter decreasing gradually in line with cortisol’s independent rhythm. Therefore, we tentatively suggest that the optimal time-point for examining the association between testosterone and brain-behavior effects with a 100-mg transdermal preparation lies between 1.5 h and 4.15 h following treatment. Our recommendations are applicable in the context of a transdermal administration route in samples with similar anthropometric characteristics (including age, gender, sexual orientation, and body mass index) as well as similar educational and ethnic backgrounds. These pharmacokinetic data provide important guidelines for the timing optimization of studies investigating the causal influence of exogenous testosterone on the brain and behavior.

## Data Availability Statement

The datasets generated for this study are available on request to the corresponding author.

## Ethics Statement

The study was approved by the local ethical committee of the Medical Faculty of the RWTH Aachen University and all subjects gave written informed consent in accordance with the Declaration of Helsinki.

## Author Contributions

AP and SR designed the study. AP supervised data acquisition and performed data analyses. AP wrote the manuscript. SR, MV, BH-D, BT, KK, and UH revised the manuscript critically for intellectual content. All authors contributed to and approved the final version of the manuscript.

## Conflict of Interest

The authors declare that the research was conducted in the absence of any commercial or financial relationships that could be construed as a potential conflict of interest.

## Footnotes

1 Serum T levels increased significantly with 5.01 nmol/L 60 minutes relative to baseline when assigning an alpha criterion level of statistical significance at p = .01, *t* *_0-60_*(119.9) = -3.92, *p*= .005, Cohen’s *d*= .834, 99% CI [0.67, 10.95].
